# Is There a Place for Anti-Thymocyte Globulin in Steroid-Refractory Acute Graft-Versus-Host Disease??

**DOI:** 10.1007/s12288-025-02099-4

**Published:** 2025-07-24

**Authors:** Adrianna Spałek, Krzysztof Woźniczka, Anna Kopińska, Kinga Boral, Anna Armatys, Helena Krzemień, Patryk Węglarz, Grzegorz Helbig

**Affiliations:** Department of Hematology and Bone Marrow Transplantation, Faculty of Medicine in Katowice, Medical University of Silesia, Katowice, Poland

**Keywords:** Acute graft-versus-host-disease, Allogeneic Hematopoietic Stem Cell Transplantation, Corticosteroid Refractoriness, Rabbit anti-thymocyte Globulin, Ruxolitinib

## Abstract

**Purpose:**

Acute graft-versus-host disease (aGVHD) remains a fatal complication after allo-HSCT. Acute GVHD resistant to corticosteroids (SR-aGVHD) and ruxolitinib (RUX) negatively affects survival and raises the question of subsequent therapeutic options.

**Methods:**

We retrospectively evaluated efficacy and toxicity of rabbit anti-thymocyte globulin (rATG, Thymoglobulin^®^, Sanofi) in 16 patients with grade III-IV aGVHD resistant to at least two prior lines of treatment (median 3), including RUX. Patients received rATG at a median total dose of 6 mg/kg (range 3.5-8 mg/kg) for 5 days.

**Results:**

Combined response (CR + PR) was demonstrated in 62.5% of patients (3-CR and 7-PR). Median overall survival (OS) for the whole group was 3.33 months (range 0.96–53.3). rATG-responders showed significantly longer 12-month OS than non-responders (*p* < 0.05). Most common adverse events during rATG treatment included CMV and BKV reactivations (*n* = 5; 31.3%), myelosuppression- grade IV thrombocytopenia (*n* = 9; 56.3%) and grade III-IV granulocytopenia (*n* = 7; 43.8%). After median follow-up of 3.9 months (range 0.96–53.3), 11 patients died. All non-responders died from aGVHD progression. Among rATG-responders, 3 patients ceased due to infectious complications and 2 because of cerebral hemorrhage.

**Conclusion:**

rATG provides satisfactory response rates in advanced SR-aGVHD failing prior treatments, however non-relapse mortality reduces survival rates among responders.

## Introduction

Acute graft-versus-host disease (aGVHD) remains a major cause of mortality after allogeneic hematopoietic stem cell transplantation (allo-HSCT). Refractoriness to systemic corticosteroids (CS) occurs in approximately 50% of patients resulting in high mortality rate reaching up to 60% in advanced disease stages [1,2]. The JAK2 inhibitor- ruxolitinib (RUX) used as a second-line therapeutic option for steroid-refractory (SR) patients, demonstrated higher overall response rate and higher duration of response when compared to control therapy in REACH2 trial [3]. Nevertheless, both REACH-2 trial and real-life data show that a significant proportion of patients will eventually lose response to RUX [4–6]. This latter group remains a real challenge for clinicians and the choice of subsequent lines of therapy depends mainly on center experience.

Rabbit anti-thymocyte globulin (rATG, Thymoglobulin^®^, Sanofi) is an immunosuppressant which can be administered for patients who failed prior treatments. Its multifaceted mechanism of action relies on T-cell depletion, modulation of key cell surface molecules that mediate endothelium interactions, induction of apoptosis in B-cell lineage or interference with dendritic cell properties [7]. Up-to-date results show variable efficacy of rATG in the treatment of SR-aGVHD with response rates between 30 and 60% and high incidence of opportunistic infections and EBV-related post-transplant lymphoproliferative disorders (PTLD) [8–10].

Herein, we report on the efficacy and safety of rATG for 16 patients with advanced aGVHD resistant to at least two prior lines of immunosuppressants (including RUX).

## Patients and Methods

This analysis included 16 patients treated with rATG as a salvage therapy for grade (G) III-IV aGVHD in our center between years 2019–2023. Data were retrospectively obtained from hospital records. All patients provided informed consent in accordance with Declaration of Helsinki. Acute GVHD was diagnosed and graded according to the Mount Sinai Acute GVHD International Consortium (MAGIC) guidelines [11]. As a first-line treatment all patients received 2 mg/kg/day of systemic methylprednisolone (MP). Steroid refractoriness was defined as follows: (1) disease progression after 3 days of therapy with 2 mg/kg per day MP, (2) failure to improve after 7 days of corticosteroids (CS) treatment with MP 2 mg/kg per day, (3) progression to a new organ after treatment with MP 1 mg/kg per day or (4) recurrence after CS taper [12]. As a second and subsequent lines of treatment patients received the following immunosuppressive agents as per decision of treating physician: RUX, mycophenolate mofetil (MMF), tacrolimus (TAC), cyclosporin A (CsA), extracorporeal photopheresis (ECP) and basiliximab. Criteria of RUX-refractoriness included: (1) progression of GVHD symptoms after 5–10 days of RUX treatment, (2) lack of improvement after at least 14 days of RUX intake, (3) loss of response at any time [12]. All patients received rATG infusion in the total dose of 3.5-8 mg/kg for 5 days. Response to rATG was evaluated between 7 and 14 days after the last day of infusion as recommended [13]. Overall responses were defined as follows: complete remission (CR)- resolution of all symptoms and laboratory manifestations attributed to GVHD; partial response (PR)- improvement in at least one organ without deterioration in others; mixed response (MR)- improvement in one or more organ with deterioration in others; no response (NR)- no change in any involved organs or progressive disease. Both MR and NR were considered as treatment failures [14]. All patients received anti-infective prophylaxis with acyclovir, trimethoprim/sulfamethoxazole and voriconazole/posaconazole. Since July 2022 for all cytomegalovirus (CMV)-seropositive recipients letermovir was implemented up to + 100 day after allo-HSCT. Due to high risk of infectious complications with multidrug resistant bacteria once weekly patients were routinely obtained throat swabs and urine cultures, according to our center procedures. Blood monitoring for CMV reactivation using quantitative PCR (polymerase chain reaction) was performed once-twice weekly up to 100 days after allo-HSCT and subsequently as needed. In case of dysuria or hematuria, a polyomavirus BK reactivation using quantitative Real-Time PCR test was carried out from blood and urine. Cytopenia was defined according to the Common Terminology Criteria for Adverse Events, Version 4.03 (CTCAE).

### Statistical Analysis

The Kaplan-Meier log-rank test was used for performing the survival curves. Statistical calculations were performed using Statistica Data Miner 13.3. The significance level of the statistical tests was considered as *p* ≤ 0.05.

## Results

Sixteen patients (12 male and 4 female) were treated with rATG as a salvage therapy for GIII-IV aGVHD resistant to at least two prior lines of immunosuppressive treatments. Median age at transplant was 42.5 years (range 20–60). The study patients received allografts for acute myeloid leukemia (AML; *n* = 9), chronic myelomonocytic leukemia (CMML; *n* = 2), blast crisis of chronic myeloid leukemia (BC-CML; *n* = 2), T-cell acute lymphoblastic leukemia (ALL-T; *n* = 1), multiple myeloma (MM; *n* = 1) and myelodysplastic syndromes (MDS; *n* = 1). Twelve patients were transplanted in CR (10 in CR1 and 2 in CR2) and four patients were in active disease. Nine patients received grafts from HLA-fully matched related donors, five from 10/10 HLA-matched unrelated donors, one from 9/10 HLA-mismatched unrelated donor and one from haploidentical donor. In total, 11 patients (68.7%) received myeloablative conditioning (MAC), whereas reduced-intensity conditioning (RIC) was provided for 5 patients (31.3%). Peripheral blood was a source of hematopoietic stem cells for all cases. For the GVHD prophylaxis patients received: CsA + methotrexate (MTX) (*n* = 8), TAC + MTX (*n* = 2), CsA + MMF (*n* = 2), TAC + MMF (*n* = 2), MMF + MTX (*n* = 1). In vivo T-cell depletion with anti-thymocyte globulin has been used for matched related transplants in recipients older than 50 years or female recipient for male donor and for unrelated stem cell transplant. Six patients received ATG Grafalon^®^ (ATG-G)– 15 mg/kg for related and 30 mg/kg for unrelated transplantation. In two cases rATG was given at a total dose of 4.5 mg/kg. For haploidentical transplantation prophylaxis with post-transplant cyclophosphamide (PT-Cy) with TAC and MMF was used (Table 1).

**Table 1:** Patient characteristics

**Patients,** ***n***	16
**Sex**,** male (%)**	12 (75)
**Age at transplant**,** median (range)**	42.5 (20–60)
**Baseline disease**,** n (%)**	
AML CMML CML-BL ALL-T MM MDS	9 (56.3) 2 (12.5) 2 (12.5) 1 (6.3) 1 (6.3) 1 (6.3)
**Disease status prior to allo-HSCT**,** n (%)**	
CR1 CR2 NR	10 (62.5) 2 (12.5) 4 (25)
**Donor type**,** n (%)**	
MRD MUD MMUD haplo-HSCT	9 (56.3) 5 (31.3) 1 (6.3) 1 (6.3)
**Donor/recipient CMV status**,** n (%)**	
positive/positive negative/negative negative/positive	11 (68.8) 2 (12.5) 3 (18.7)
**Conditioning regimens**,** n (%)**	
myeloablative reduced intensity	11 (68.7) 5 (31.3)
**Source of stem cells**,** peripheral blood**,** n (%)**	16 (100)
**GVHD prophylaxis**,** n (%)**	
CsA + MTX TAC + MTX CsA + MMF TAC + MMF MMF + MTX PT-Cy + MMF + TAC	8 (50) 2 (12.5) 2 (12.5) 2 (12.5) 1 (6.3) 1 (6.3)
**ANC > 0.5 (x10**^**9**^**/L)**,** median (range)**	16.5 (12–26)
**PLT > 20 (x10**^**9**^**/L)**,** median (range)**	14 (12–26)

ALL-T -T-cell acute lymphoblastic leukemia, allo-HSCT-allogeneic hematopoietic stem cell transplantation, AML-acute myeloid leukemia, ANC-absolute neutrophil count, CML-BL-chronic myeloid leukemia blast crisis, CMML-chronic myelomonocytic leukemia, CMV-cytomegalovirus, CR-complete remission, CsA-cyclosporin A, GVHD-graft-versus-host disease, haplo-HSCT-haploidentical hematopoietic stem cell transplant, MDS-myelodysplastic syndrome, MM-multiple myeloma, MMF-mycophenolate mofetil, MMUD-mismatched unrelated donor, MRD-fully matched related donor, MTX-methotrexate, MUD-matched unrelated donor, NR-no remission, PLT-platelet count, PT-Cy-post-transplant cyclophosphamide, TAC-tacrolimus

Ten patients (62.5%) developed GIII and six (37.5%) GIV aGVHD. All patients had GIII-IV gut involvement. Six patients presented liver manifestation as well. Fourteen out of sixteen patients presented skin aGVHD. Median time from allo-HSCT to aGVHD occurrence was 52 days (range 31–212). rATG was implemented after median of 3 ineffective lines of immunosuppressive therapies (range 2–4). First-line treatment in all cases consisted of systemic MP at dose of 2 mg/kg. Once steroid resistance has been confirmed, patients received subsequent therapeutic options: MMF (*n* = 13), RUX (*n* = 8), TAC (*n* = 4), CsA (*n* = 3), ECP (*n* = 1) and basiliximab (*n* = 1). Doses of CsA and TAC were adjusted to achieve a therapeutic level.

**Fig. 1:**
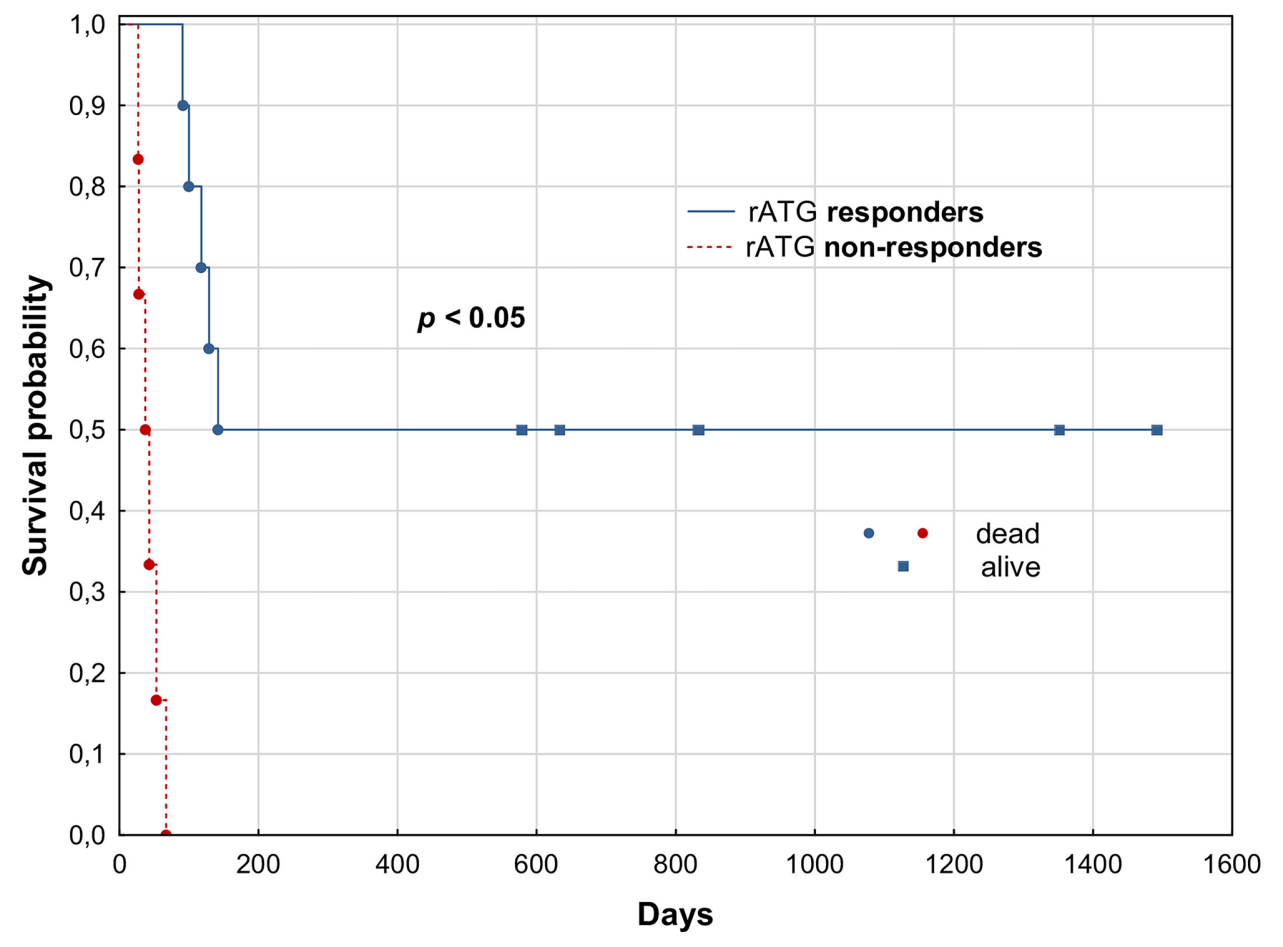
Survival curves for rATG responders and non-responders

The median total dose of rATG was 6 mg/kg (range 3.5-8 mg/kg). rATG was administrated consecutively for 5 days. The overall response rate (ORR) was 62.5% (10/16 patients), including 3 patients in CR and 7 in PR. Among eight patients previously treated with RUX, four responded to rATG treatment. Median overall survival (OS) for the whole group was 3.3 months (range 0.96–53.3). rATG-responders showed significantly longer 12-month OS than non-responders (50% vs. 0%; *p =* 0.004). Overall response rates (CR + PR) were comparable between those with and without ATG for GVHD prophylaxis. Survival curves for each group are presented in Fig. 1. Among most common adverse events during rATG therapy were grade IV thrombocytopenia (*n* = 9) and grade III-IV granulocytopenia (*n* = 7). CMV reactivation occurred in 3 patients and 2 patients developed hemorrhagic cystitis due to BK polyomavirus reactivation. Two other patients suffered from pneumonia related to *Pneumocystis Jiroveci*. No fungal infections were observed. In 4 patients chronic GVHD as a continuum of aGVHD developed.

After median follow-up of 3.9 months (range 0.96–53.3) from aGVHD onset 11 patients died (all 6 patients from non-responders group and 5 patients from the responders group). All the non-responders died to the rapid progression of GVHD symptoms with gastrointestinal obstruction or hepatorenal syndrome. Patients from rATG-responders group died from infectious complications (*n* = 3) - *Kl. Pneumoniae* sepsis, *SARS-CoV-2* and *Clostridioides Difficile* or intra-cerebral hemorrhage (*n* = 2) (Table 2).

**Table 2:** GVHD response to rATG treatment

**Interval between transplant and onset of aGVHD, days; median (range)**	52 (31–212)
**aGVHD grade, ** ***n *** **(%)**	
grade III grade IV	10 (62.5) 6 (37.5)
**Prior GVHD therapies; median (range)**	3 (2–4)
**Prior immunosuppressive drugs; n (%)**	
methylprednisolone MMF RUX tacrolimus cyclosporin ECP basiliximab	16 (100) 13 (81.3) 8 (50) 4 (25) 3 (18.8) 1 (6.3) 1 (6.3)
**Total dose of rATG (mg/kg); median (range)**	6 (3.5-8)
**rATG dose; n (%)**	
2–4 mg/kg > 4 mg/kg	2 (12.5) 14 (87.5)
**Overall response**,** n (%)**	
CR PR NR	3 (18.8) 7 (43.8) 6 (37.5)
**Median follow-up from aGVHD onset**,** months; median (range)**	3.9 (1-53.5)
**Alive at last contact**,** n (%)**	5 (31.3)
**Causes of death**,** n (%)**	
GVHD progression infection intra-cerebral hemorrhage	6 (37.5) 3 (18.8) 2 (13)

aGVHD-acute graft-versus-host-disease, CR-complete remission, ECP-extracorporeal photopheresis, MMF-mycophenolate mofetil, NR-no response, PR-partial remission, rATG-rabbit anti-thymocyte globulin Thymoglobulin^®^, RUX-ruxolitinib

## Discussion

The use of rATG in treatment of SR-aGVHD has already been attempted in early 2000s. It was demonstrated that ~ 30–60% of SR-aGVHD patients achieved response to rATG, however high risk of opportunistic infections and development of EBV-PTLD posed serious limitations of its widespread use [8–10]. Reported rates of serious bacterial infections during rATG treatment reach 20–37% whereas systemic fungal infections occur in 32% of steroid-resistant patients [8–10]. PTLD development may affect even one in four patients [8]. Since RUX was successfully introduced for steroid-refractory patients, the use of rATG has been significantly reduced. It seems rationale to find a balance between the dose of rATG and the risk of serious adverse events.

It was showed that 2.5 mg/kg of rATG used for GI-IV aGVHD resulted in comparable response rate to doses of 10-15 mg/kg previously administrated in steroid-resistant patients [8, 14]. Moreover, doses less than 2 mg/kg significantly diminished 1-year non-relapse mortality (NRM) when compared to doses ≥ 2 mg/kg (62% vs. 82%) [14]. Other factors which may influence NRM included older age, GIV aGVHD, liver involvement and lack of response to the rATG therapy.

Favorable outcome of very low-dose rATG has been confirmed recently in a short communication by *Yamada et al.* [13]. 13 patients received with rATG at 0.5-1.5 mg/kg as a second line treatment for GII-IV aGVHD achieving overall best response rate in 77% of treated patients. Although 1-year OS reached only 27%, with high treatment-related mortality at 54%, the results were favorable when compared to high-dose rATG. Interestingly, for patients with liver involvement very low-dose rATG was found to be utterly ineffective.

Our data confirm the high efficacy of rATG at approximately 60% which stands with previous reports. However, unlikely to previous papers, our patients received rATG after at least two unsuccessful lines of treatment, and we focused only on patients with the most advanced III-IV grades of aGVHD. It should be also pointed out that 50% of our study population received rATG after prior RUX failure. Taking that all into consideration our results are encouraging. The responses were observed regardless of initial organ involvement- 50% for skin and liver manifestations and 62.5% for intestinal involvement. However, despite satisfactory results, infectious complications negatively affected OS resulting in NRM of 30% among patients who responded to rATG infusion. Overall, 56.3% patients had viral or bacterial infections during treatment leading to death in 3 cases. High incidence of infections may be enhanced by prolonged immunosuppression period due to many different lines of treatment. Interestingly, only 3/16 patients presented CMV reactivation. This may be explained by the prophylactic use of letermovir that significantly reduced CMV reactivation incidence to single cases. It should be also noted that we used rATG doses > 2 mg/kg, what in the context of previously mentioned papers, may negatively affect infectious complications incidence [13,14].

In this regard, the availability of new anti-viral prophylaxis (e.g. letermovir for CMV reactivation) or anti-fungal drugs as well as antibiotics seems to be essential part of the early stage of treatment. Nevertheless, rATG used as a last-chance treatment resulted in aGVHD resolution in five patients. None of the survivors developed PTLD or relapsed during follow-up.

The question regarding current place for rATG in the therapy of SR-aGVHD remains open. In the era of RUX, it seems that it should be used at the earliest after RUX ineffectiveness [4–6]. There are no unequivocal recommendations for optimal treatment after RUX failure. The only available real-life report on 48 RUX-resistant patients showed that even 30% of patients died prior to the initiation of subsequent therapy with low chance for obtaining response [4]. Yet presented study groups are too small to draw conclusions regarding effectiveness of different agents.

## Conclusions

rATG can remain an effective therapeutic option for salvage therapy of SR-aGVHD for RUX-resistant patients. Nevertheless, it should be bear in mind that long-term survival is low due to high incidence of fatal infectious complications. Application of lower than standard doses of rATG may remain a valuable option, however more studies are needed.

## Data Availability

Study data are available with the authors and can be shared upon reasonable request.
